# A modified pull-through approach with a pedicled bone flap for oral and oropharyngeal cancer resection: a feasibility study

**DOI:** 10.1007/s00276-024-03302-3

**Published:** 2024-02-15

**Authors:** Norbert Neckel, Peter H. Neckel, Bernhard Hirt, Christian Doll, Elena Hofmann, Susanne Nahles, Max Heiland, Kilian Kreutzer, Steffen Koerdt

**Affiliations:** 1 Department of Oral and Maxillofacial Surgery, Charité – Universitätsmedizin Berlin, corporate member of Freie Universität Berlin, Humboldt-Universität zu Berlin, and Berlin Institute of Health, Augustenburger Platz 1, Hindenburgdamm 30, 13353 Berlin, Germany; 2 Institute of Clinical Anatomy and Cell Analysis, University of Tübingen, Tübingen, Germany

**Keywords:** Oral cancer, oral squamous cell carcinoma (OSCC), Oropharyngeal cancer, oropharyngeal aqumous cell carcinoma (OPSCC), Resection, Pull-through, Mandibular lingual release, Mandibular split

## Abstract

**Purpose:**

Compromised swallowing, speaking, and local complications are the major disadvantages of established approaches to the posterior tongue and oropharynx. The mandibular split involves an esthetically unpleasant bipartition of the lower lip and is prone to bony non-union or sequestration. The conventional pull-through technique on the other hand lacks the secure reattachment of the lingually released soft tissues.

**Methods:**

The feasibility of a new modified pull-through approach was tested on three anatomical specimens. CAD/CAM cutting guides were used to design a retentive bone flap to properly refixate the genioglossus and geniohyoid muscles after the procedure. The radiographic assessment and treatment planning was performed on 12 cadavers. The entire procedure was tested surgically via dissection in three of those cases. This procedure was then applied in a clinical case.

**Results:**

Precise repositioning and dynamic compression of bony segments was possible reproducibly and without injury to adjacent structures. In all dissected cases, a median lingual foramen was found and in two cases vessels entering it could be dissected Radiologic anatomical landmarks were sufficient in all 12 cases to perform the clinical planning procedure. Clinically, the osteotomized segment demonstrated good blood supply and plateless repositioning was verified postoperatively via cone beam scan.

**Conclusion:**

The method presented is safe and easy to perform. Individual cutting guides improve the safety and accuracy of the procedure, potentially eliminating the need for osteosynthesis. We provide the anatomical and radiologic basis for clinical evaluation of this pedicled bone flap procedure and present the clinical application of this modified pull-through approach.

**Supplementary Information:**

The online version contains supplementary material available at 10.1007/s00276-024-03302-3.

## Introduction

Head and neck cancer therapy frequently has burdensome side effects, which can result from the surgical therapy itself or the multimodal treatment concept, including adjuvant radiotherapy or chemoradiotherapy. The consequent impairment in speaking and swallowing is a major factor in lowering patient’s quality of life (QoL), even after tumor eradication [1]. Tumors in the posterior third of the oral cavity, at the base of the tongue, or oropharyngeal primaries are a surgical challenge because the conventional transoral approach may be inadequate. Because transoral robotic surgery (TORS) is available in few specialized centers, the prevailing techniques used in these situations include pull-through (PT) or mandibular lingual release (MLR) and mandibular lip-split (MS), all of which have side effects and pose potential risks [2, 3]. For MS in particular, these include altered occlusion and injury of adjacent teeth and impairment of bony consolidation, especially in patients who received postoperative radiotherapy [3, 4]. Another disadvantage is the requirement for titanium plates to fixate the repositioned mandibular segments, which can compromise radiation therapy, provoke tissue irritation and is prone to infections [5, 6]. Studies by Cheng et al. and Li et al. have revealed that the PT approach seems to be beneficial in terms of esthetics, time of surgery, postoperative complications, pain, and patient-related QoL [3, 4]. By contrast, Devine et al. reported a better outcome of MS regarding speech, swallowing, and chewing [7]. Nevertheless, both surgical techniques are associated with the risk of impaired tongue movement, as well as difficulty in swallowing and speaking [3, 7]. Cheng et al. [3] valuated both functions 6 months after T4a tongue and floor of mouth cancer resections and found that 35 of 58 MLR and 24 of 33 MS patients still depended on tube feeding and only 29 of 58 MLR as well as 24 of 33 MS patients demonstrated good speech intelligibility [3]. Furthermore, postoperative tracheostomy maintenance, an indicator for the risk of aspiration, exceeded one month in 31 of 58 MS and 19 of 33 MS patients. The fact that the tumor resection itself, depending on its location, leads to impairment of oral function calls for a surgical technique that avoids further debilitation. Given that Devine et al. [7] for instance included almost exclusively T1 and T2 tumors of the anterior oral cavity when comparing MLR and MS, one has to acknowledge the limited comparability of existing studies on the topic [7]. Nevertheless, the results of this study on small anteriorly located tumors unmask the potentially detrimental effect of the traditional MLR on QoL in terms of speech, swallowing and chewing [7].

The aims of this study are to combine the advantages of both surgical approaches, to provide adequate access to the posterior oral cavity, ensure secure anterior refixation of the tongue, and preserve mandible continuity without the necessity for osteosynthetic plating.

In this study, we present a modified PT approach, which comprises CAD/CAM-guided mental osteotomy of the mandible to preserve the attachments of the genioglossus (GGM) and geniohyoideus (GHM) muscles on the mandible. This pedicled, retentive bone flap can be repositioned into its socket after tumor resection resulting in preserving anterior tongue fixation. The findings of this study demonstrate the feasibility of this novel surgical approach and provide the basis for further clinical evaluation.

## Materials and methods

### Body donors

The surgical procedure performed on three body donors at the Institute of Clinical Anatomy & Cell Analysis. The causes of death according to the certificate of death were stroke, cardiac infarction, and cardiac failure, respectively. No morphological abnormalities, scars, or deformities were visible externally. One body donor had a central venous catheter, which did not affect the surgical area. The body donors gave informed consent to use their cadavers for research purposes in accordance with the Declaration of Helsinki. The local ethics committees approved the anatomical procedure (Project Nr. 081/2022BO2) and clinical application (EA2/305/20).

### Fixation

Embalming was performed by intra-arterial injection by the femoral artery using an IJT-50 injection system (Thalheimer, Ellwangen, Germany). Depending on the condition of the cadaver’s vascular system, we used a perfusion pressure of 0.5–1.0 bar, with an ethanol-based fixative solution consisting of 71.3% (v/v) ethanol (Roth Carl Roth GmbH + Co. KG, Karlsruhe, Germany), and 24.5% (v/v) glycerol (Roth) in water.

### Photo and video documentation

We photo-documented relevant surgical steps under standardized conditions using a Nikon D300 camera equipped with a Vario lens. We used the integrated FullHD camera (1920 px × 1080 px) of two marLED X operating lights (KLS Martin, Tuttlingen, Germany) for video documentation.

### Artwork

Figures including Artwork were created using Procreate 5.3.5 (Savage Interactive, Hobart, Australia), CorelDraw X7, Version 17.6.0.1021 (Corel Corporation, Ottawa, Canada), and Affinity Photo 1.10 (Serif (Europe) Ltd, Nottingham, United Kingdom). Video editing was performed using DaVinci Resolve 18 (Blackmagic Design Pty Ltd., Port Melbourne, Australia).

### Surgical approach

The surgical procedure was performed according to a standardized protocol. Computer tomography scans of all three anatomical specimens were performed, with a slice thickness of 1.25 mm. These were used for the virtual planning and design of the cutting guides.

**Fig. 1:**
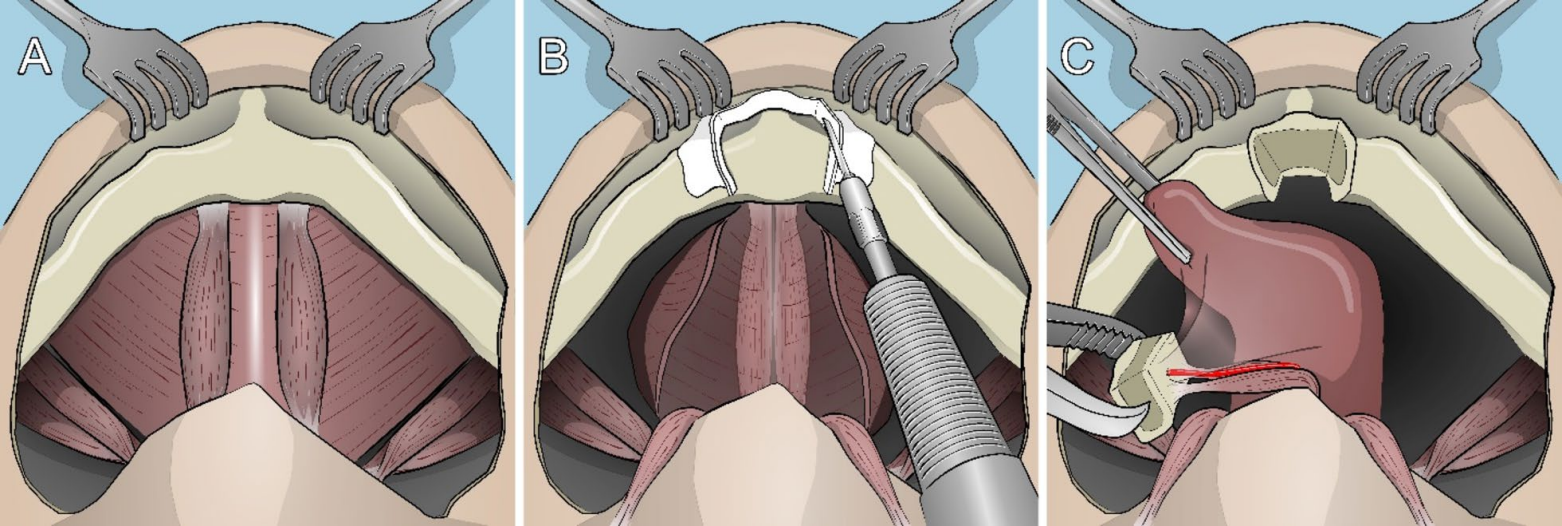
(**A**–**C**) Schematic illustration of the planned surgical procedure, view from below. (**A**) Initial situation after transection of the platysma. (**B**) Osteotomy after the detachment of the digastric muscles and lateral aspects of the mylohyoid muscle with the customized cutting guide in situ. (**C**) The modified pull-through approach, with the genioglossus (transparent) and geniohyoid muscles attached to the bony segment, including the medial lingual artery (in red)

**Fig. 2:**
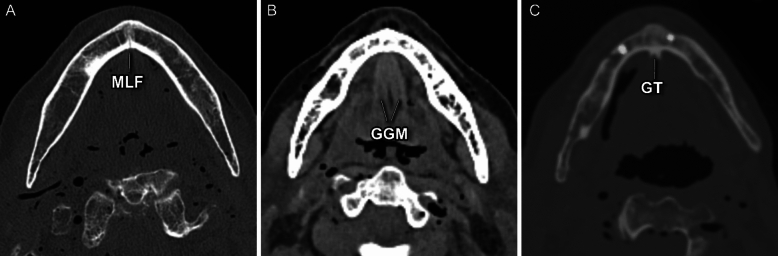
(**A**–**C**) CT morphometric illustrations of the anatomical landmarks for the design of the cutting guides: (**A**) Medial lingual foramen (MLF), (**B**) genioglossus muscle (GGM) in soft tissue fenestration and (**C**) the genial tubercle (GT)

**Fig. 3:**
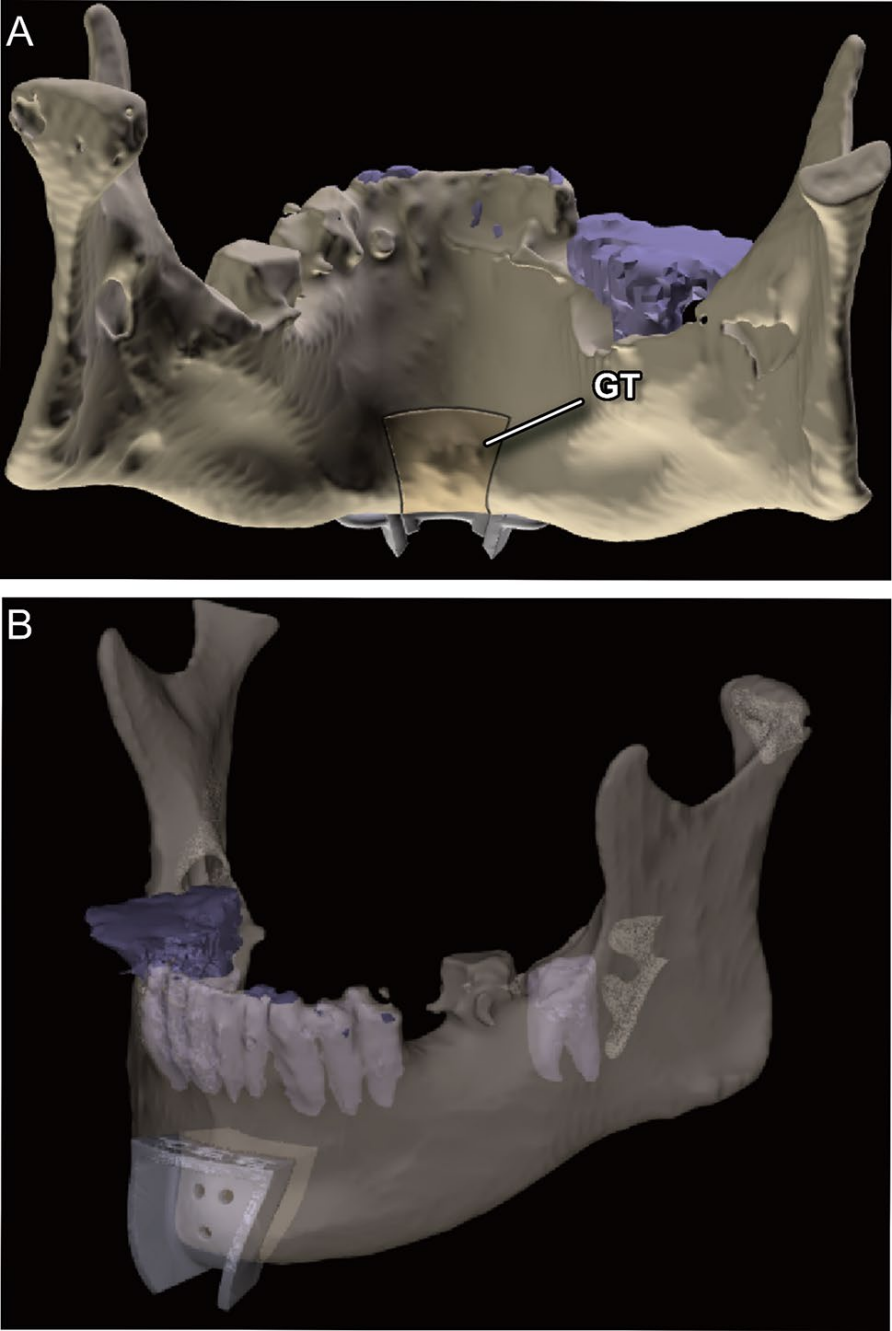
(**A**–**B**) Exemplary three-dimensional renderings of a mandible with the corresponding cutting guide design: (**A**) Visualization of the genial tubercle and the surrounding osteotomy lines and (**B**) Transparent visualization of the cutting guide with the bony segment, respecting the location of the dental roots

**Fig. 4:**
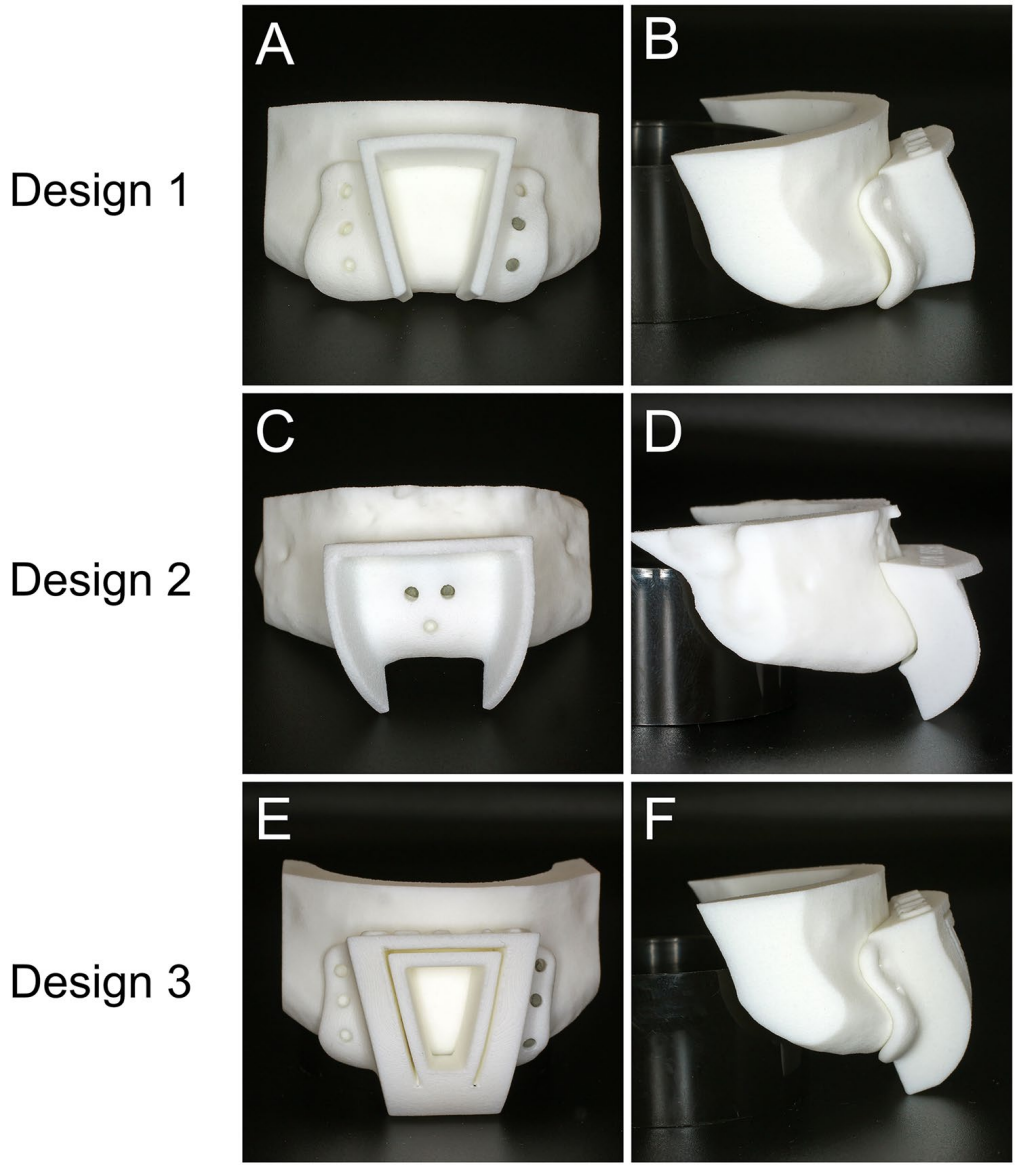
(**A**–**F**) Illustrations of cutting-guide designs

**Fig. 5:**
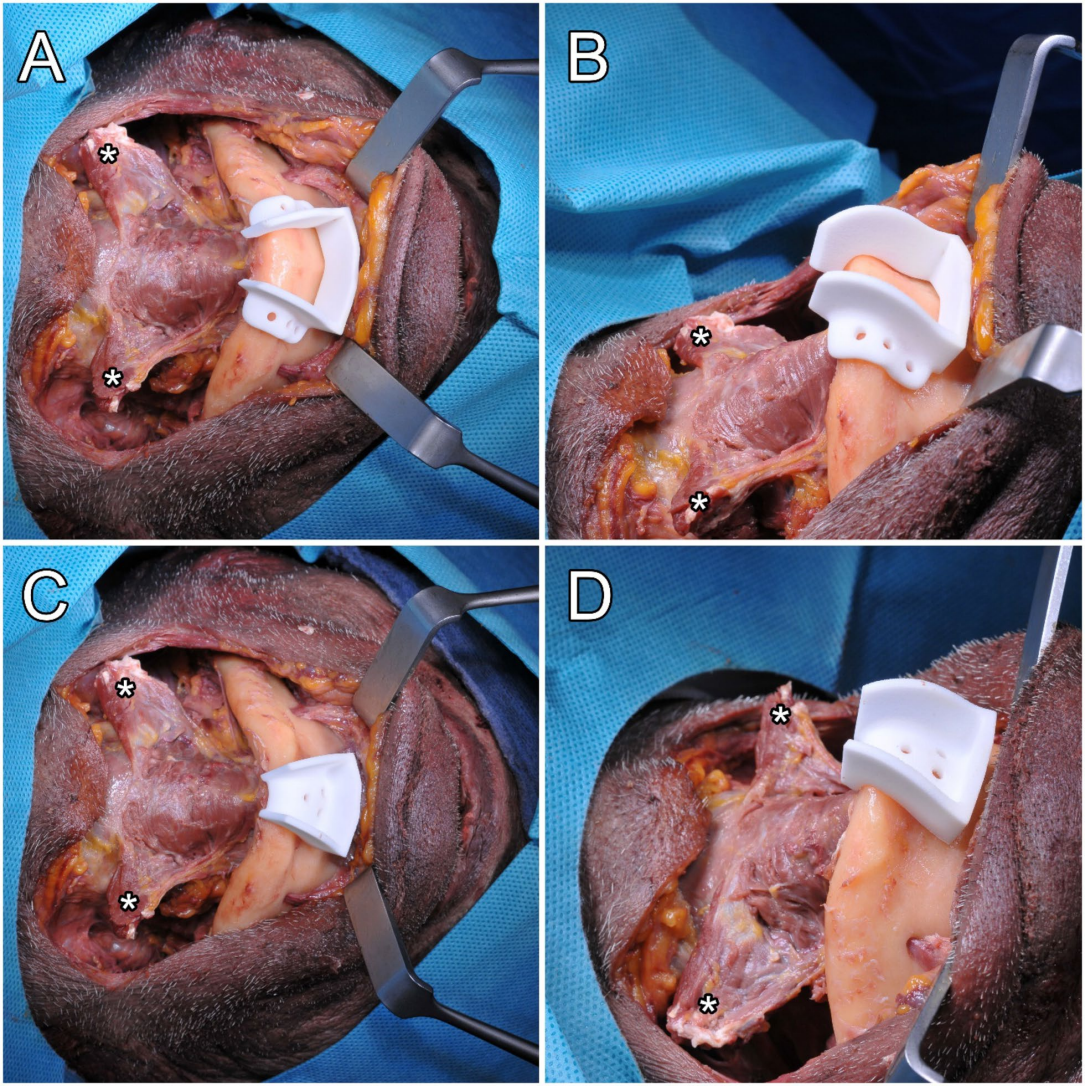
(**A**–**D**) Cutting guides (designs A and B) in situ (*detached attachment of the anterior bellies of the digastric muscles)

**Fig. 6:**
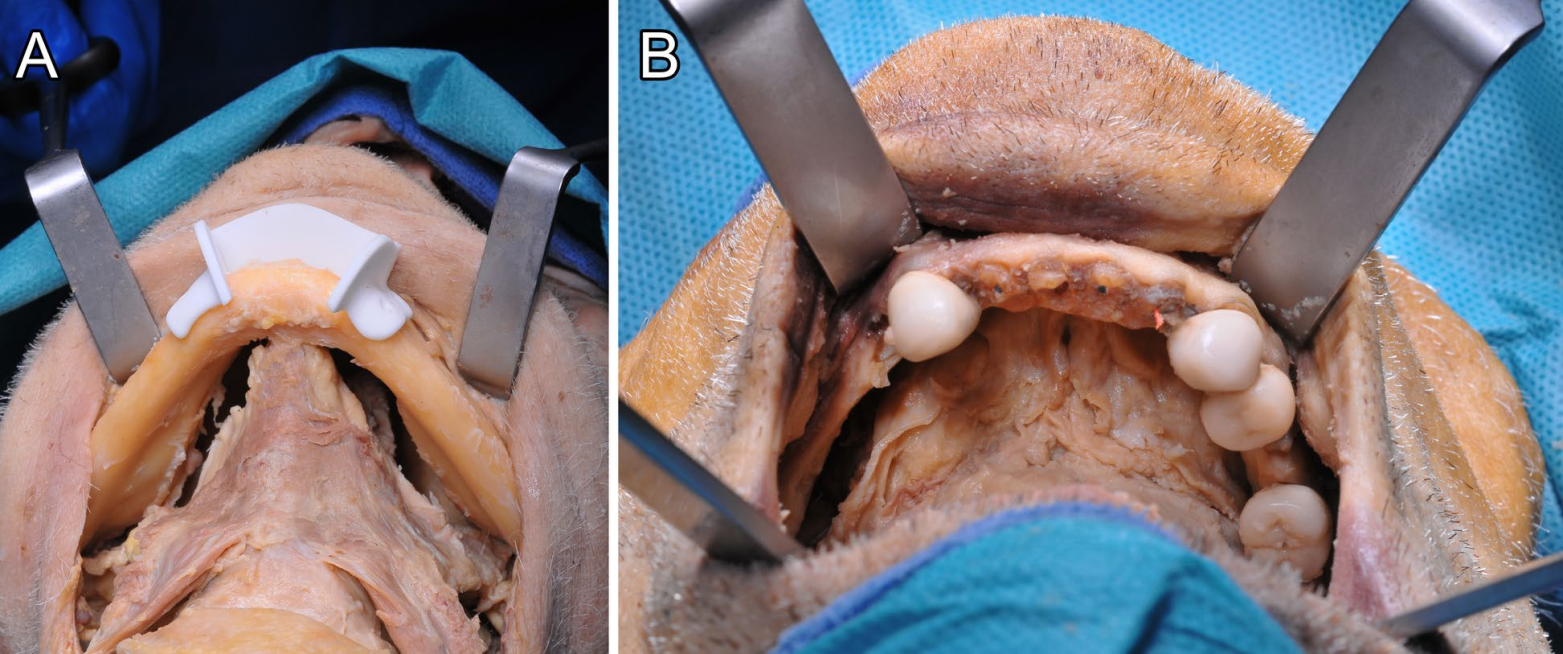
(**A**–**B**) Attachment of genioglossus (GGM) and geniohyoideus (GHM) from the submandibular view (**A**) and after lingual release from the intraoral view (**B**) after the detachment of mylohyoid and digastric muscles from the mandible

The cutting guides provide the generation of a pyramid- or wedge-shaped bony fragment that would allow self-fixation due to the tapering cutout and the pulling force applied by the intact musculature (Figs. 1, 2, 3, 4, 5 and 6).

Surgery was performed under standard conditions. A bilateral neck incision was made in the skin fold, > 2 cm below the inferior mandibular border. A skin platysma flap was raised and ROBINS levels IA and IB dissection was performed. Then, an incision was made along the lower mandibular border to detach the vestibular lateral and anterior periosteum, including the mentalis muscle, to gain access to the mental region. Furthermore, the mylohyoid muscle was detached from the lingual aspects of the posterior mandible. If no interference with the cutting guide occurred, the median aspect of the mylohyoid could be left attached to the mandible. Subsequently, a crestal or linguo-marginal incision was made to detach, elevate, and protect soft tissue before the osteotomy. Then the cutting guides were positioned and fixed with screws. An oscillating saw was used to perform the osteotomy. The bony segment was mobilized anteriorly upon mouth opening and brought out of its socket. Lastly, the tongue was mobilized and its base pulled into view. The bony segment was then repositioned into the socket.

The entire procedure was filmed from two angles and photodocumented. The procedure is illustrated in Fig. 1.

### Design of the cutting guides

Cutting guides for the V-shaped osteotomy of the anterior mandibular border were designed and manufactured using CAD/CAM technology. Mimics Medical 23.0 (Materialise, Leuven, Belgium) was used to segment the DICOM-Data and render 3D models of every single specimen. The cutting guides were planned and designed using Freeform plus (Geomagic, Morrisville, North Carolina). To assure the attachment of the GGM and GHM to the bony segment, the osteotomy lines were determined by the surgeon under consideration of the anatomical landmarks, such as the mental spines, the medial lingual foramina, and the GGM using bone as well as soft tissue fenestration (Fig. 2). 3D rendering of the mandibles provided additional three-dimensional orientation (Fig. 3). KLS Martin GmbH (Tuttlingen, Germany) performed the technical execution and printing (printer: FORMIGA P 110 Velocis and material: PA 2200—Polyamide 12, EOS GmbH Electro Optical Systems, Krailing, Germany). Three designs were used (Fig. 4). All cutting guide types were first positioned to assess their fit and applicability (as illustrated in Fig. 5). In each case, the subjectively best fitting guide was used to perform the osteotomy.

### Clinical application

The clinical application of this approach was planned, utilizing the anatomical landmarks in a 66-year-old patient with a cT2 cN0 squamous cell carcinoma of the left posterior border of the tongue and lower palatoglossal arch. Due to the involvement of the base of the tongue and unspecific enlargement of a contralateral node in Level IIA, a bilateral functional neck dissection (Level I-III) was performed. “Design 1” cutting guides were used to osteotomize the bony segment. The modified pull-through approach was performed as described above and the segment was repositioned without fixation with plates after reconstruction of the defect with a radial forearm flap. A postoperative CT scan was done to verify the correct repositioning of the segment.

## Results

### Cutting guides

In practical use, design 1 (Fig. 4a and b) has the advantage of reproducibility and easy positioning of the cutting guide on the mandibular vestibular aspect. Design 2 (Fig. 4c and d) offers the advantage of reduced dissection of vestibular soft tissue and periosteum laterally and facilitates the ventral removal of the bony fragment. However, positioning of the cutting guide is more challenging. Design 3 (Fig. 4e and f) offers a facilitated angulation of osteotomy lines; however, its bulky design has many disadvantages regarding in-surgery handling and lack of saw cooling. Therefore, guide 3 was excluded from application in this study.

Feasibility of the planning procedure and reproducibility according to the anatomical landmarks (mental spines, medial lingual foramina and GGM) were assessed on 12 CT scans of anatomical specimens. This included three cadavers in which the entire procedure was tested (Table 1). In every case, at least two landmarks were definable, offering sufficient information for the procedure. The modified PT would not have been a viable option in five specimens due to an edentulous, highly atrophic mandible. The male/female ratio of the anatomical specimens was 1.4. The age distribution is illustrated in Table 1.

**Table 1:** Analysis of the definability of the anatomical landmarks (mental spine, median lingual foramen (MLF) and genioglossus muscle (GGM)). Five patients presented edentulous and highly atrophic (e + a) mandibles. The mean age (years) at the time of death was 87.6 years. D 1 and D 2 indicate the respective cutting guide designs 1 and 2 that were used for the osteotomy

Specimens	Age	Mental spine	MLF	GGM	
A320^a^	81	No	Yes	Yes	D 1
A331^a^	89	Yes	Yes	Yes	D 2
AL179^a^	89	Yes	No	Yes	D 2
A342	92	Yes	Yes	Yes	e + a
A343	93	Yes	Yes	Yes	
A344	81	Yes	Yes	Yes	
A345	91	Yes	Yes	Yes	e + a
A346	73	Yes	Yes	Yes	e + a
A347	92	Yes	No	Yes	e + a
A349	83	Yes	Yes	Yes	
A350	92	Yes	Yes	Yes	
A341	95	Yes	Yes	Yes	e + a
***n* = 12**	**87.6**	**91,7%**	**83.3%**	**100%**	

^a^Were included into the presented anatomical dissection

### Osteotomy

For reproducible osteotomies, assurance of retentive design, and thinnest osteotomy lines possible, we evaluated various saws available for mandibular resection. Although, piezo-osteotomy is safe and reliable, we preferred using a delicate jigsaw (Osseo scalpel, Saw blade GD320 13/0.3/0.3 mm, AESCULAP®, Aesculap AG, Tuttlingen, Germany), because its cutting thickness (0.30 mm) is lesser than that of the slender piezo saw (0.35 mm), ensuring better fragment retention after repositioning at the end.

### Surgical procedure

**Fig. 7:**
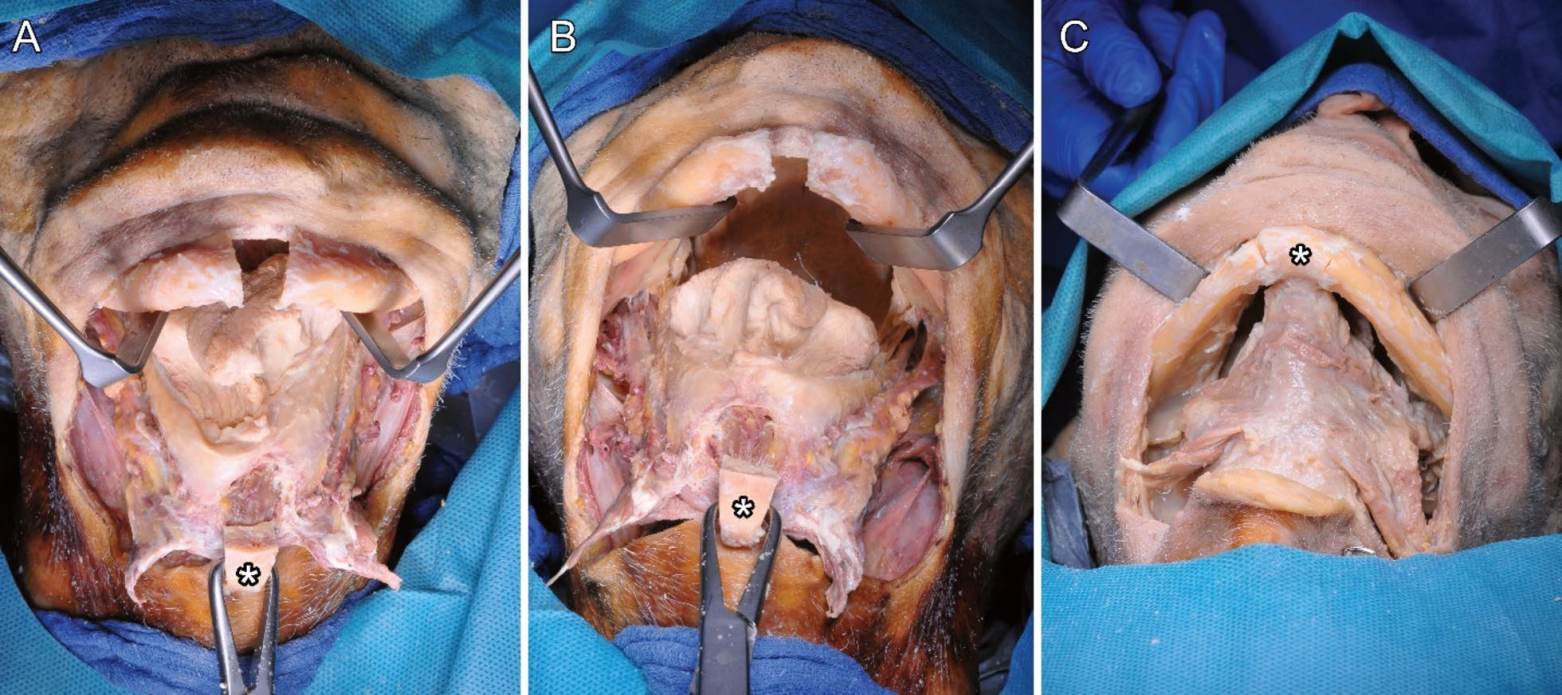
(**A**–**C**) Completed surgical access with anterior V-shaped osteotomy (*) from cranial (**A**) and submandibular (**B**) views. Repositioning of the fragment (**C**)

After the mandible was exposed using the bilateral submandibular approach and neck dissection for lymph node clearance ROBINS levels IA and IB, incision of the periosteum at the lower mandibular border and exposure of the mandible, including bilateral exposure of the mental nerve, was performed, followed by guided anterior osteotomy (Figs. 5, 6 and 7).

**Fig. 8:**
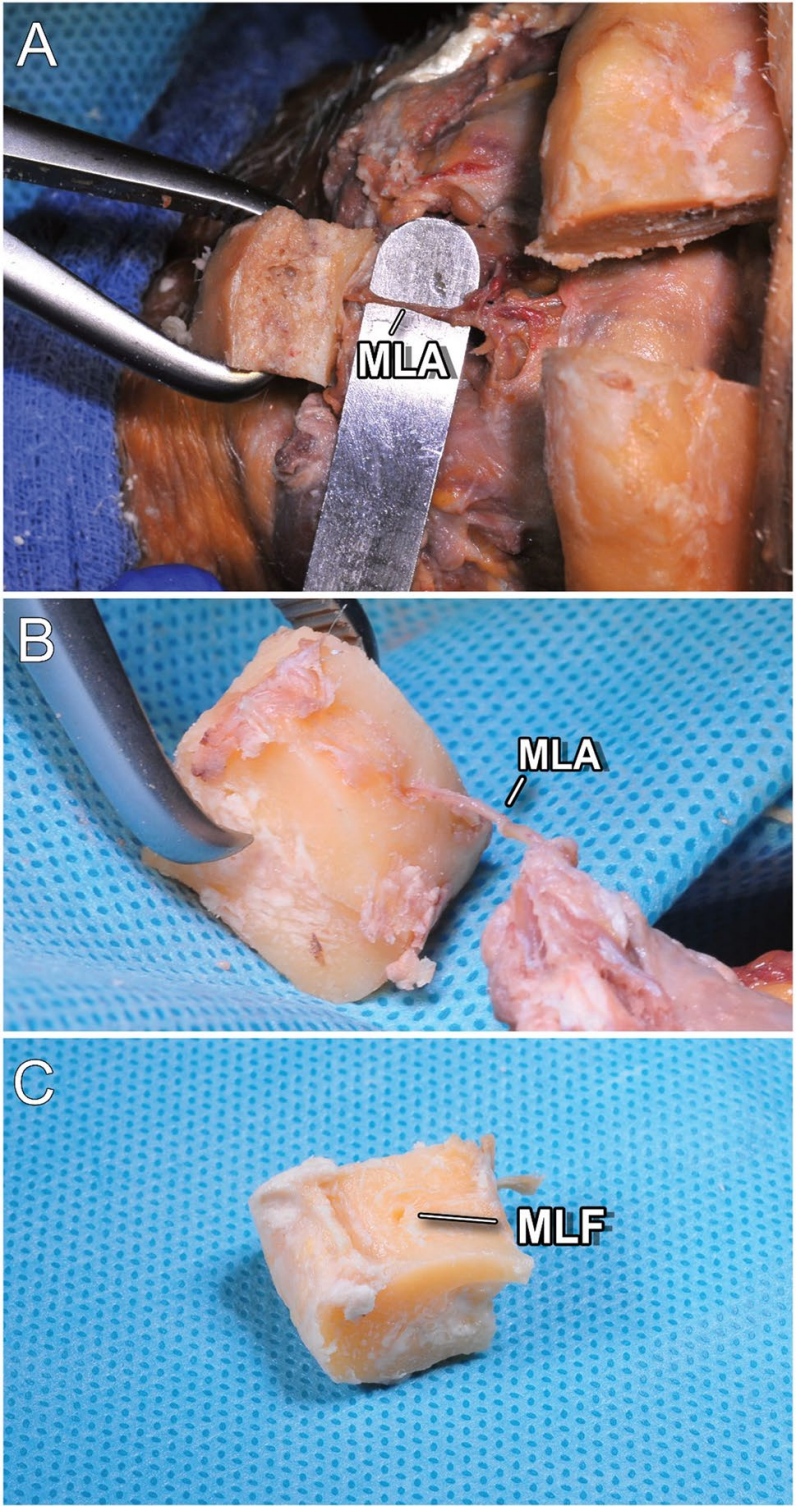
(**A**–**C**) Medial lingual foramen (MLF) with the corresponding medial lingual artery (MLA)

**Fig. 9:**
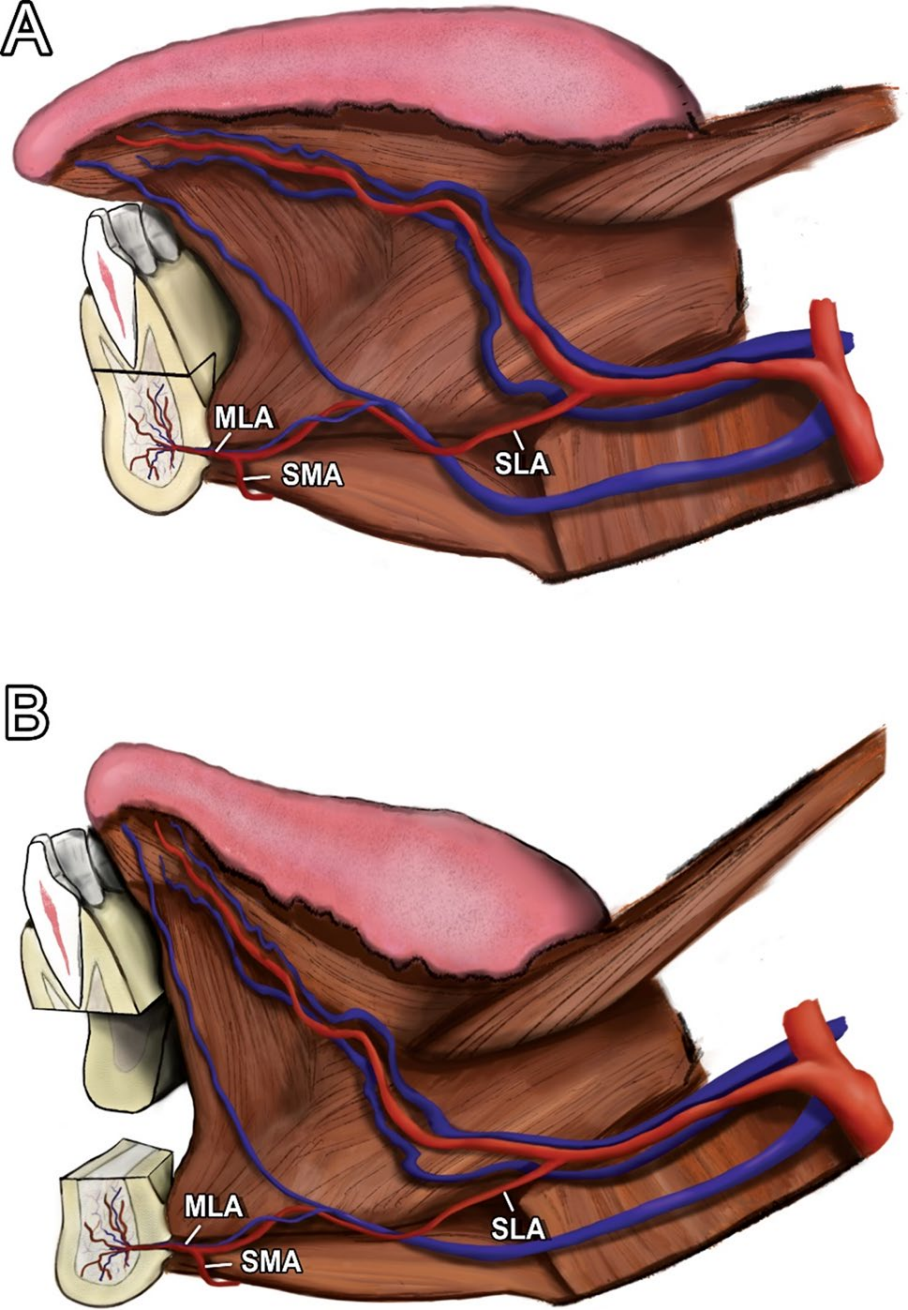
(**A**–**B**) Sagittal view demonstrating the sublingual artery (SLA), submental artery (SMA), and medial lingual artery (MLA) before mobilization (**A**) and after detachment of the bony segment (**B**)

The MLF was present in all three cases (Fig. 8a–c). Additionally, we dissected and demonstrated vessels entering the MLF in two cases (Fig. 8a and b). For simplicity, we referred to these vessels as “medial lingual artery” (MLA) in this study. The anatomical drawing in Figs. 9a and b illustrates the blood supply.

Dynamic compression of the bony segment could be simulated by closing the mouth of the specimens despite the lack of muscle tone (supplemental video).

### Clinical application

**Fig. 10:**
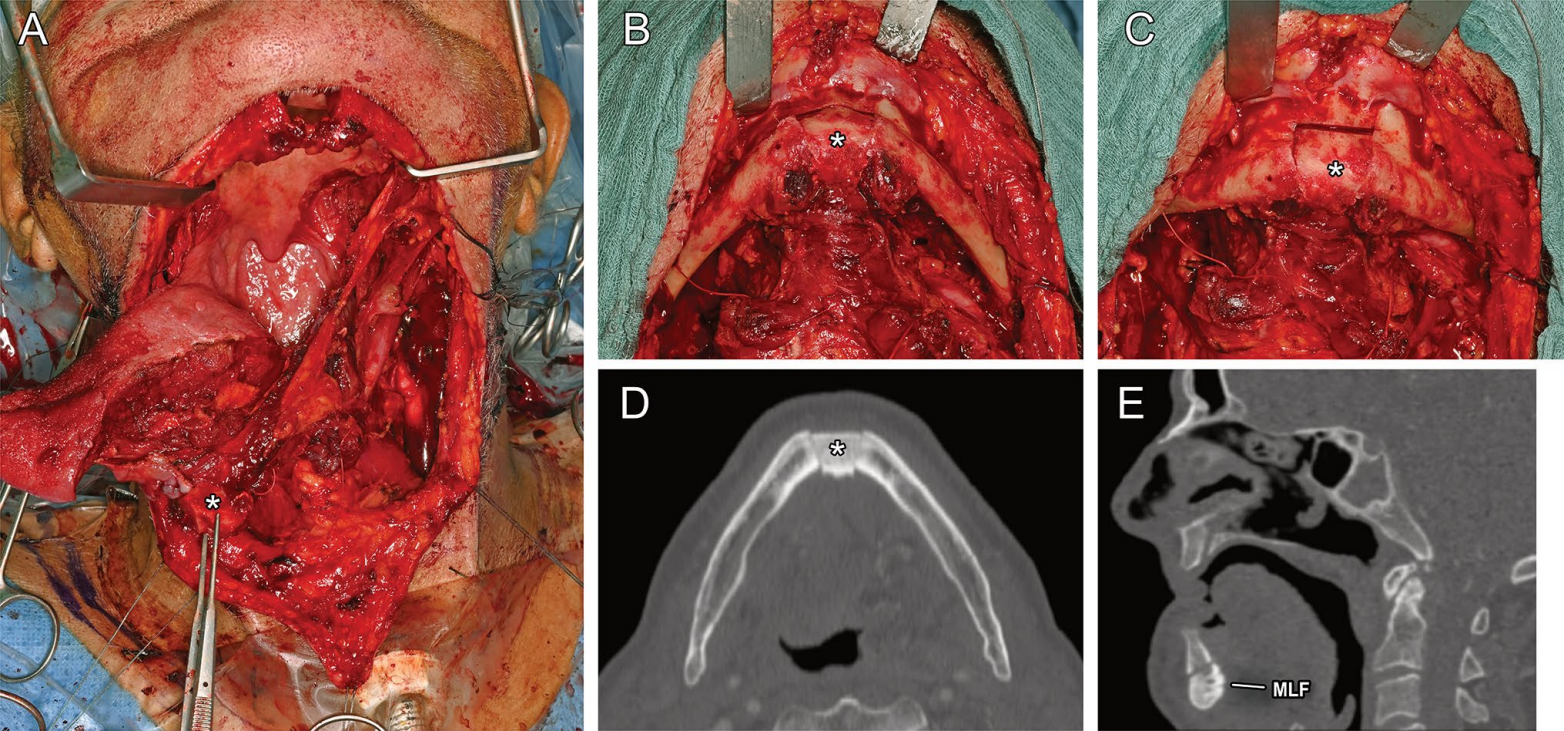
(**A**–**E**) Graphical synopsis of the clinical application demonstrating the modified pull-through approach after tumor resection (**A**) and after repositioning of the bony segment (in axial view: **B** and frontal view: **C**), which is marked with asterisks. (**D**) and (**E**) depict the CT scan 13 days after tumor resection and free flap reconstruction, demonstrating good repositioning of the bony segment and sufficient posterior airway space. MLF in E indicates the medial lingual foramen including the medial lingual canal

The tumor resection and free flap reconstruction was performed via the modified pull-through approach resulting in histopathologically negative margins of at least 5 mm without node involvement (pT2, pN0 (0/42), R0, L0, V0, Pn1). The cutting guide could be positioned stably, and the presence of the medial lingual artery could be verified via Doppler sonography. The osteotomized segment showed good perfusion and spontaneous bleeding from the osteotomized pedicled segment could be observed (supplemental video 2). After tumor resection and reconstruction of the defect, precise and stable repositioning of the segment into its socket was possible (Fig. 10). Therefore, no plating to fixate the segment was needed. The postoperative CT scan confirmed the precise repositioning and sufficient posterior airway space due to anterior fixation of the tongue (Fig. 10). On the 7th postoperative day, the tracheal cannula could be removed due to sufficient swallowing. Furthermore, the feeding tube was no longer tolerated by the patient and was removed the same day. Sufficient calorie intake was possible via smooth/pureed and high caloric diet. The patient’s speech was slightly affected by the altered anatomy after partial tongue resection, but still clear enough to understand. On day 13 after surgery, the patient was discharged from the hospital.

## Discussion

Malignancies in the posterior third of the oral cavity, base of the tongue, and oropharyngeal primaries pose a major surgical challenge. To facilitate free resection margins and adequate exposure for reconstruction, e.g., using microvascular transplants, the transoral approach must be rejected, requiring alternative approaches that compromise functional outcome and patient QoL [1]. TORS can be a valid alternative; however, its availability is limited and poor applicability in cases of larger tumors, especially with related trismus, must be considered [8]. This study aims to provide a novel feasible approach that allows adequate access while minimizing patient debilitation.

GGM, the largest and strongest extrinsic tongue muscle, plays an essential role in tongue mobility, thereby allowing complex voluntary movements, such as the oral preparatory phase of swallowing or speaking [9]. The non-voluntary pharyngeal phase of swallowing is characterized by hyolaryngeal elevation, which is promoted by the mylohyoid and GHM [9]. Because achieving the correct refixation of the mylohyoid challenging, due to its fanned-out shape, GHM integrity is important for preserving hyolaryngeal elevation [9, 10].

MLR is characterized by the detachment of both GGM and GHM; reapproximation can only be performed through the use of sutures [3, 11]. This adaptation might be inadequate, because patients subconsciously activate the involved musculature immediately after surgery, which explains the clinical and functional limitations that can be observed with traditional MLR [7].

Therefore, keeping the functionality of both GGM and GHM intact is a pivotal step to optimize swallowing and speaking after MLR and involves proper refixation of the GGM and GHM to the mandible.

GGM advancement procedures and their technical variations are used for treating obstructive sleep apnea surgically [12, 13]. The aim is to apply traction to the GGM by advancing a bony mandibular segment, including the genial tubercle, and to fixate it in an anterior position through plating [12]. This effectively prevents the base of the tongue from collapsing into the pharynx occluding the posterior airway space [13]. The use of virtually planned cutting guides is recommended for reducing the risk of failure or injury due to the close proximity to adjacent teeth and lack of visibility on the lingual aspect [12]. Moreover, the precise location of the genial tubercle cannot be predicted reproducibly due to anatomical variations [14]. However, despite its varying shape, some form of mental spine can be found in roughly 98% of the cases [15]. In concert with the MLF, which according to Wang et al. [16] could be identified radiographically in 97% of the cases and the GGM the area of interest could be identified reproducibly [16].

In our study, no injury to relevant structures or tooth roots could be observed. The workflow of virtual design and 3D-printing of cutting guides is increasingly integrated in orthognathic surgery and head and neck reconstructive oncology and will likely prevail in future [14, 17, 18].

Therefore, the clinically established procedure of GGM advancement was modified to fulfill the specific requirements of this PT approach.

To account for the anatomical vectors of force, designing a retentive shape is required (Figs. 3, 4, 5, 6). Notably, before the osteotomy, GGM and GHM force vectors are directed anteriorly and superiorly, which reversely forces the bony segment posteriorly and inferiorly after osteotomy [10].

Consequently, proper virtual planning is pivotal to avoid injury to anatomical structures and assure stable and wiggle-free repositioning of the segment, thereby preventing it from being pulled posteriorly and inferiorly. Hence, we believe plating can be unnecessary if the bone cuts are performed with a very delicate saw. This leads to the transformation of tensile forces of the muscles to compressive forces to the osteotomy lines, analogous to “natural strain of compression” described by Champy et al. [19].

Therefore, immediate postoperative muscle activation and logopedic functional exercise should be possible and even advantageous. Still, a specific regimen for postoperative care must be carefully established, not to jeopardize the potential benefit of this technique.

The findings of this study revealed that the segments could be repositioned stably and could not be elevated from their position when the mouth of the anatomical specimen was closed (supplemental video 1). This simulates the forces applied when activating GGM and GHM. Avoiding the application of osteosynthesis material is desirable, because it can cause severe complications, as seen in MS [3]. However, mandibular continuity is intact and if stable repositioning cannot be achieved, a delicate miniplate should suffice. A highly atrophic mandible is a potential contraindication. In these cases, mandibular continuity cannot be preserved or the fracture risk is excessive. The relatively large number of edentulous and highly atrophic mandibles in this cohort may arise from the high average age of almost 90 years.

Another anatomical foundation includes adequate blood supply to the bony segment to avoid sequestration and ensure postoperative consolidation. Studies have reported the presence of a lingual foramen and the corresponding canal in the midline of the symphysis in 97–100% of cases [16, 20]. These common canals can be detected through three-dimensional radiographic imaging despite having diameters ranging from 0.25 to 1.60 mm [16, 21]. Interestingly, although the respective foramina were described several times, they were named inconsistently throughout the literature [22]. Here, we have used *medial lingual foramen* (MLF) based on the nomenclature proposed by Tagaya et al. [23] and Nakajima et al. [22]. Despite contradicting reports on the presence of nerves and venous vessels in the corresponding medial lingual canals, a broad consensus exists that an arterial branch of the sublingual or submental artery passes through the MLF [20, 24, 25].

This study found that MLF was present in all three cases; in two cases, the *medial lingual artery* (MLA) could be dissected (Fig. 8a–c). In these two cases, the MLA ran protected between the bellies of GGM and GHM. According to the literature, it is supplied by the submental and sublingual artery; however, many anastomoses between the lingual, facial, submental, sublingual, and the inferior alveolar arteries have been described [22, 26, 27]. Therefore, MLA is probably fed by the sublingual rete arteriosum to which the facial as well as the lingual artery contribute [22]. Another source of supply for the bony segment includes the intact lingual periosteum and muscle attachment, which could compensate for the rare but potential lack of an MLF [28].

Considering the constant existence of MLF, a stable blood supply to the bony segment is likely. Due to its location central to mental spines, the associated vessel inserts are protected between the bellies of GGM and GHM (Figs. 8 and 9). By leaving this lingual muscle cuff as well as the lingual periosteum of the segment intact, we have demonstrated a technique that facilitates the preservation of pedicled blood supply. Hence, it can be considered a pedicled bone flap. Owing to the delicacy of MLA, this approach may prevent shearing of the vessel. Considering that GGM and GHM are separated for genioglossus advancement, it must be assumed that this specific blood supply can be compromised during this procedure [14, 29]. However, reproducible success of the procedure may be explained by random pattern supply through the muscle and periosteum [12, 29]. In contrast to that in orthognathic surgery, optimal blood supply of the segment is even more important because of the frequent necessity of adjuvant radiotherapy in oral and oropharyngeal cancer [30]. By contrast, the bony segment is positioned far from the tumor bed and a minimized local effect can be achieved by the steep dose gradient of modern radiotherapy techniques, which reduces the risk of sequestration of the segment further [30].

Potential downsides of this work derive from its nature as a study on anatomical specimens resulting in limited transferability to live patients, especially concerning the potential functional benefits of this technique.

The adequate anterior refixation of GGM and GHM as major protagonists for tongue and hyoid movement, and hence, swallowing and speaking will likely ameliorate the functional outcome in patients. Improvements of these functions would accelerate decannulation, independent food intake, social interaction and reintegration, and shortened hospitalization, consequently improving patient QoL. A modification in terms of a unilateral approach leaving the contralateral floor of the mouth attached is also conceivable. We have justified further evaluation of this modified PT approach in clinical studies.

## Conclusion

In this feasibility study, we demonstrated that the method presented is safe and easy to perform. The use of individually fabricated cutting guides minimizes the risk of injury to dental roots or disruption of muscle insertions. Proper osteotomy design and thin cuts enable the precise repositioning of the bony segment, thereby potentially eliminating the need for the implantation of osteosynthetic material. Furthermore, we found anatomical evidence to consider it a pedicled bone flap procedure. Therefore, we provide the anatomical and clinical basis for further evaluation of this modified pull-through approach in clinical studies, which must address potential risks and evaluate the proper postoperative care for functional optimization and osseous reintegration of the bony segment.

### Supplementary Information

Below is the link to the electronic supplementary material.

Supplementary file1 Video 1 demonstrating the pedicled bone flap and its repositioning into its bony socket (MP4 113141 KB)

Supplementary file2 Video 2 demonstrating the presence of the median lingual artery (MLA) and good perfusion of the osteotomized bony segment (MP4 43650 KB)

## Data Availability

The data that support the findings of this study are available from the corresponding author upon reasonable request.
